# The History and Capabilities of Herbal Simples

**Published:** 1894-06-23

**Authors:** W. T. Fernie


### The History and Capabilities of Herbal Simples.

By W. T. Fernie, M.D. *(Continued from page 420, Vol. XIV.)*

### HEMLOCK AND HENBANE.

"Robert and Richard were two pretty men; they lay in bed till the clock struck ten," says an ancient rhyme. So the hemlock and the henbane are two poisonous plants which are only bestirred into our notice as simples for outside uses. Nevertheless, the former herb is common enough in English hedgerows, and the latter is by no means rare. The hemlock is the handsomest of our umbelliferous plants, growing to four or five feet in height, with delicate leaves most elegantly cut, and with a hollow stem, powdered blue at its bottom, and marked conspicuously by purple blotches and spots. The rough chervil resembles it in being likewise spotted, but this is very hairy, and its stem is swollen beneath each joint. The name hemlock, or homlock, is derived from the haulms, or dry hollow stalks of the plant after flowering, called kecksies in Essex, and giving rise to the old saying, "as hollow as a kix." Thus also they are called "wode-whistle," "being of no use except for poor men's fuel and children's play." The herb in all its parts is highly soporific; and on this account witches were thought to have a special liking for it. Socrates is said to have been poisoned with a draught concocted from this plant. Coles says, "If asses chance to feed much upon hemlock they will fall so fast asleep that they will seem to be dead." The witches in "Macbeth" made the "root of hemlock, digged in the dark," an ingredient of their hellish broth. Botanically, the plant is named Conium, from the Greek *κονος*, a top, the whirling motion of which resembles the giddiness produced in the human brain by a poisonous dose of the juice. This juice, if taken internally, paralyses the spinal cord; but when used locally as a poultice it admirably soothes pain in cancer, and ulcers, whilst the vapour from the bruised leaves and stalks, when steamed in boiling water, will quickly relieve any spasmodic bronchial cough. The action of the plant depends on its alkaloids, coniine and methyl coniine, which are best obtained from the mature but green fruit. The plant should be gathered for its juices when beginning to flower, and it is beyond question that by the virtues of these when applied as a dressing the condition of open sores regarded as cancer may be much improved. Likewise many glandular swellings reputed to be of a cancerous nature may be made to disappear by a similar local use of the fresh-bruised hemlock plant. Conium plasters were formerly commended for drying up the milk, and are now found of service for subduing nervous palpitation of the heart. Our druggists dispense hemlock juice, combined with some spirit of wine, for preserving it. They also keep an extract of hemlock, mixed with potash, for evolving the vapour of the herb. If twenty drops of this are placed on a sponge in an inhaler over boiling water so that the medicated steam is inspired through the mouth and nose signal relief will be obtained in whooping cough and for spasmodic bronchial asthma. The dried leaves of hemlock, if put into a small bag and boiled in water for a few minutes, being then applied hot to a gouty part, will immediately abate the pain. Furthermore, they will help to soften and dissolve the hard concretions which form about gouty joints. Under proper medical advice the extract and the juice of hemlock may be most beneficially administered in cancer and as a soporific. The black henbane in Gerarde's day grew almost everywhere by highways, in the borders of fields, about dung-hills, and untoiled places. But now it has become much less common as a rustic herb in England. We find it occasionally in railway cuttings and in rubbish on waste places. The plant is large and dull of aspect, with woolly sea-green leaves, and bearing straw-coloured, bell-shaped flowers, painted with purple lines. It has an oppressive heavy odour, and is rather clammy to the touch. This herb is also called hog's-bean, and its botanical name—Hyoscyamus—means likewise the bean of the hog, which animal eats it with impunity.

